# A sensitive and specific assay to characterize plasma kallikrein activity in plasma from patients with hereditary angioedema^☆^

**DOI:** 10.1016/j.waojou.2025.101135

**Published:** 2025-11-13

**Authors:** Daniel K. Lee, Arije Ghannam, Nivetha Murugesan, Denis Vincent, Micaela Dona, Danny M. Cohn, Adil Adatia, Michael D. Smith, Paul K. Audhya, Sally L. Hampton, Edward P. Feener

**Affiliations:** a KalVista Pharmaceuticals Inc, Framingham, MA, USA; b KalVista Pharmaceuticals Limited, Salisbury, United Kingdom; c KininBio, Grenoble, France; d Université de Montpellier, Montpellier, France; e Centre de Compétence des Angioedèmes à Bradykinine, Montpellier, France; f Allergist, Private Practice, Paris, France; g Amsterdam University Medical Center, University of Amsterdam, Amsterdam, the Netherlands; h University of Alberta, Edmonton, AB, Canada

**Keywords:** HAE, Hereditary angioedema, Plasma kallikrein, Biomarker

## Abstract

**Introduction:**

Plasma kallikrein (PKa) activity is increased in the plasma of patients with hereditary angioedema (HAE) and has been implicated in other kallikrein-kinin system (KKS)–mediated diseases. Exogenous substrates commonly used in PKa assays can be cleaved by multiple plasma proteases, which reduce assay specificity and sensitivity for PKa. We describe a sensitive and specific assay to detect PKa activity in plasma as a candidate biomarker for HAE.

**Methods:**

PKa activity was measured in plasma samples from patients with HAE with decreased C1 inhibitor (C1INH) levels or activity who were not receiving prophylactic medications for HAE (HAE-C1INH; *n* = 25), from individuals with a presumptive diagnosis of HAE with normal C1INH (HAE-nC1INH; *n* = 3), and from age-matched controls without HAE (*n* = 57). Samples were analyzed at baseline and after 6 h of cold incubation at 4 °C. Amidolytic activity was measured in the absence and presence of a PKa-specific inhibitor (KV999272). Specific PKa (sPKa) activity was quantified by the subtraction of amidolytic activity not inhibited by KV999272 from the total measured amidolytic activity.

**Results:**

In control plasma, sPKa activity was 0.69 ± 0.07 nmol/min/mL at baseline and 0.88 ± 0.11 nmol/min/mL after 6 h of cold incubation (mean ± SEM, *p* = 0.0062); the 95th percentile of sPKa activity was 1.87 nmol/min/mL at baseline and 3.07 nmol/min/mL after cold incubation. In plasma from patients with HAE-C1INH, sPKa activity was 3.43 ± 0.64 nmol/min/mL at baseline and 24.53 ± 8.92 nmol/min/mL after 6 h of cold incubation (*p* = 0.023). sPKa activity in HAE-C1INH plasma samples was above the 95th percentile for control plasma with assay sensitivity of 84% and specificity of 95%. The area under the receiver operating characteristic curve was 0.98 (*p* < 0.0001). sPKa activity in all plasma samples from patients with HAE-nC1INH was above the 95th percentile for control plasma after 6 h of cold incubation.

**Conclusion:**

We developed a specific PKa assay that can detect low levels of PKa activity in plasma and can differentiate patients with HAE-C1INH from controls without HAE with high sensitivity and specificity. Using this assay, we demonstrated that sPKa activity is elevated during the intercritical period in patients with HAE-C1INH and in those with HAE-nC1INH compared with controls when measured after 6 h of cold incubation. This sensitive and specific PKa assay could be useful to characterize PKa activity in plasma samples from patients with HAE and could potentially serve as a future candidate biomarker for HAE-nC1INH.

## Introduction

Hereditary angioedema (HAE) is a rare genetic disease that causes unpredictable recurrent episodes of disfiguring or disabling swelling in cutaneous and submucosal tissues.^1^ These attacks occur spontaneously in a variety of locations, typically affecting the intestine, face, extremities, and genitalia, and can be life threatening when the upper airway is involved.^2^ Swelling during HAE attacks is mediated by elevated levels of bradykinin, resulting in increased vascular permeability in the affected tissue.^1^^,^^3^ Bradykinin is a vasoactive peptide that is generated by plasma kallikrein (PKa)-mediated cleavage of high molecular weight kininogen (HK).^4^^,^^5^ Excessive bradykinin generation in HAE is caused by the dysregulation of the kallikrein-kinin system (KKS).^4^^,^^5^

C1 inhibitor (C1INH), a serine protease inhibitor (SERPIN), is the primary inhibitor of KKS proteases PKa and factor XIIa (FXIIa).^4^^,^^6^^,^^7^ A deficiency in C1INH function allows uncontrolled PKa and FXIIa protease activity, thereby increasing bradykinin generation and leading to HAE attacks.^4^^,^^5^ The most prevalent and well-studied cause of HAE is C1INH deficiency caused by mutations in the *SERPING1* gene that result in decreased levels or activity of the C1INH protein.^5^ HAE caused by decreased levels (type 1) or activity (type 2) of C1INH (HAE-C1INH) is typically diagnosed by laboratory assays for C1INH antigenic level and function.^5^ HAE-C1INH can be further characterized by analyses of the genetic defects in the *SERPING1* gene.^5^^,^^8^^,^^9^ More than 800 variants in the *SERPING1* gene have been identified in patients with HAE-C1INH.^10^

In addition, HAE with significant morbidity can occur in the presence of normal C1INH activity and levels (HAE-nC1INH).^11^^,^^12^ Given the lack of defining biomarkers, the presumptive diagnosis of HAE-nC1INH requires exclusion of alternate causes and determination of responsiveness to medications (such as antihistamines, corticosteroids, and B2 bradykinin receptor antagonists), which can result in diagnostic delays.^13^^,^^14^ Mutations have been identified in multiple genes that can modulate the KKS, thereby enabling the identification of subtypes of HAE-nC1INH in a limited number of cases.^14^, ^15^, ^16^, ^17^, ^18^, ^19^, ^20^, ^21^, ^22^ However, the link between some of these mutations and dysregulation of the KKS is not fully understood and the genetic causes in most patients with a presumed HAE-nC1INH diagnosis remain unknown.^23^^,^^24^ While case reports provide some evidence that HAE-nC1INH is responsive to KKS inhibitors,^14^^,^^25^, ^26^, ^27^, ^28^, ^29^, ^30^ biomarkers to characterize PKa activity, which may contribute to HAE attacks, in patients with HAE-nC1INH are currently an unmet clinical need.

Multiple methods to assess KKS activation have been used to characterize HAE plasma, although few appear to be used in clinical practice.^31^^,^^32^ These methods include measurements of PKa substrates and products, including HK and bradykinin and their fragments,^33^^,^^34^ and measurements of PKa activity.^35^ Immunoassays and mass spectrometric methods have been used to quantify cleaved HK and bradykinin,^36^^,^^37^ whereas PKa enzyme assays directly measure catalytic activity.^38^^,^^39^ While each method has its advantages and limitations,^40^ we sought to develop and optimize an assay to specifically quantify PKa activity without the need for highly specialized reagents and instrumentation.

We have developed a sensitive and specific measurement of PKa activity in plasma and quantified specific PKa (sPKa) activity in plasma from a cohort of patients with confirmed HAE type 1 or type 2 who were not receiving prophylactic therapies for HAE and from a control group of volunteers without HAE. This assay may enable further characterization of the KKS in HAE-C1INH, HAE-nC1INH, and other KKS-mediated diseases, and may help predict response to currently available treatment options.

## Methods

### Study participants

Control whole blood samples were obtained from volunteers without HAE from the United Kingdom and United States, including 23 samples sourced from REPROCELL (Beltsville, MD, USA). All volunteers were screened using a self-reported medical history questionnaire to establish that the control individuals had no history of symptoms of angioedema.

For the HAE-C1INH cohort, whole blood samples from adults with a confirmed diagnosis of HAE-C1INH were collected during the intercritical period (i.e., between HAE attacks) as a pre-dose sample in the open-label pharmacokinetic part 1 of a phase 2 trial evaluating sebetralstat (NCT04208412), as described previously.^41^ As described in the enrollment criteria of the study, these patients did not receive long-term prophylactic therapies for HAE and had 3 attacks during the 93-day screening period before blood collection. The number of days since the last acute treatment ranged from 9 to 69.

Blood samples were also obtained from 3 patients with a presumed diagnosis of HAE-nC1INH and a history of responding to HAE medications. Patient 1 with HAE-nC1INH (HAE-nC1-S1) was a 22-year-old female with a history of subcutaneous and facial edema and previous treatment with tranexamic acid and plasma-derived C1 esterase inhibitor (Berinert; CSL Behring, Melbourne, Australia). Genetic testing for F12 mutation was negative. Patient 2 with HAE-nC1INH (HAE-nC1-S2) was a 62-year-old male with a history of lip and tongue edema and previous treatment with tranexamic acid; genetic testing was not performed. Patient 3 with HAE-nC1INH (HAE-nC1-S3) was a 45-year-old female with a history of subcutaneous edema with facial and abdominal attacks and previous treatment with tranexamic acid and Berinert. Testing was performed for *F12, PLG, CPN1, HS3ST6, MYOF, KNG1, ANGPT1* mutations; no known mutation found. None of the patients had been trialed on omalizumab. Samples from patients 1 and 2 were collected during the intercritical period. Two samples were collected from patient 3: 1 during the intercritical period, and 1 during an HAE attack. The age range and sex distribution for participants in this study are provided in Table 1.

**Table 1:** Demographic information for volunteers without HAE (controls) and for patients with HAE-C1INH and HAE-nC1INH.

Sample	Age, range, years	Sex, %	
		Female	Male
Controls (*n* = 57)	23–65	39	61
HAE-C1INH (*n* = 25)	19–68	64	36

**HAE-nC1INH (*n* = 3)**	**Age, years**	**Sex**	
HAE-nC1-S1	22	Female	
HAE-nC1-S2	62	Male	
HAE-nC1-S3	45	Female	

HAE, hereditary angioedema; HAE-C1INH, hereditary angioedema with decreased levels or activity of C1 inhibitor; HAE-nC1INH, hereditary angioedema with normal levels and activity of C1 inhibitor; HAE-nC1-S1, patient 1 with HAE-nC1INH; HAE-nC1-S2, patient 2 with HAE-nC1INH; HAE-nC1-S3, patient 3 with HAE-nC1INH

### Ethics, consent and permissions

Collection of samples was approved by relevant institutional review board or ethics committees, followed good clinical practice guidelines, observed the Declaration of Helsinki, and followed the guidance of the International Conference on Harmonization. All participants provided written informed consent.

### Sample collection and pre-analytical conditions

To prevent exogenous activation of the KKS,^42^ samples of whole blood were collected in plastic collection tubes containing 3.2% sodium-citrate and were kept at room temperature for no longer than 48 h prior to centrifugation at 2000*g* for 10 min at 20 °C for control and HAE-nC1INH samples, which is the recommended protocol. The HAE-C1INH samples were obtained from part 1 of the phase 2 clinical study of sebetralstat and were collected in 3.2% sodium citrate collection tubes and centrifuged at 1500*g* for 10 min at 4 °C.^41^ Plasma was extracted and immediately frozen and stored at −80 °C until analysis.

### sPKa activity assay

Frozen plasma samples were thawed on ice prior to use. Immediately upon thawing, samples were aliquoted into 0.2-mL PCR strips and buried in wet ice (4 °C) for 6 h of cold exposure. For time course experiments, 4- and 12-h time points of cold exposure were also analyzed. Samples were also measured at baseline, prior to the cold exposure protocol.

Measurements of PKa enzyme activity (V_max_) were made using the chromogenic substrate H-D-Pro-Phe-Arg-*p*NA·2HCl (Bachem, Torrance, CA, USA). Prior to each measurement, samples were incubated with either the selective PKa inhibitor KV999272^43^ (also referred to as FE999272 or VA999272, final concentration 1 μM) or phosphate-buffered saline (PBS) for 15 min at room temperature. Samples were then diluted 1:20 in assay buffer (50 mM Tris, 150 mM NaCl, pH 7.8) containing the chromogenic substrate, and enzyme amidolytic activity was determined by measuring the rate of *p*NA formation as an increase in absorbance at 405 nm at 30 °C over 15 min using a Tecan Spark microplate reader (Tecan, Morrisville, NC, USA). The maximum rate (V_max_) was calculated from the absorbance data using Excel and expressed in nanomoles per minute per milliliter.

To calculate sPKa activity, the following equation was used, whereby “total activity” was activity observed in samples assayed without inhibitor KV999272; “amidolytic activity not inhibited by a PKa inhibitor” was activity observed in samples assayed with inhibitor KV999272.

Total activity – amidolytic activity not inhibited by a PKa inhibitor = sPKa activity

Low QC (human pooled control plasma; Visucon-F, Affinity Biologicals, Ontario, Canada) and high QC (human C1 esterase inhibitor depleted plasma (Innovative Research, Novi, MI, USA) samples were included on each assay plate and total amidolytic activity measured in the presence and absence of substrate. The assay was deemed successful if the blank subtracted activity for the low QC was <25% CV and the high QC variability was <5% CV.

The effect of the broad-spectrum protease inhibitor 4-(2-aminoethyl) benzenesulfonyl fluoride hydrochloride (AEBSF, Sigma-Aldrich, Burlington, MA, USA) was analyzed in 4 control samples from volunteers without HAE for which less than 40% of their amidolytic activity was inhibited by KV999272. These samples underwent cold incubation for 6 h, after which they were incubated with AEBSF (final concentration 2 mM), KV999272 (final concentration 1 μM), or PBS for 15 min at room temperature, prior to measurement of activity.

### Statistics

Results are expressed as mean ± SEM. Data analysis was performed using GraphPad Prism, version 10.0.2 (GraphPad Software, San Diego, CA, USA). All pairwise comparisons were performed using a 2-tailed, paired *t* tests; *p* values <0.05 were considered statistically significant.

The performance of the sPKa activity assay was evaluated by calculating area under the curve (AUC) of the receiver operating characteristic (ROC) curve. A threshold was set by calculating the 95th percentile of control samples after 6 h of cold incubation.

## Results

### Cold exposure increased amidolytic activity in plasma

**Fig. 1:**
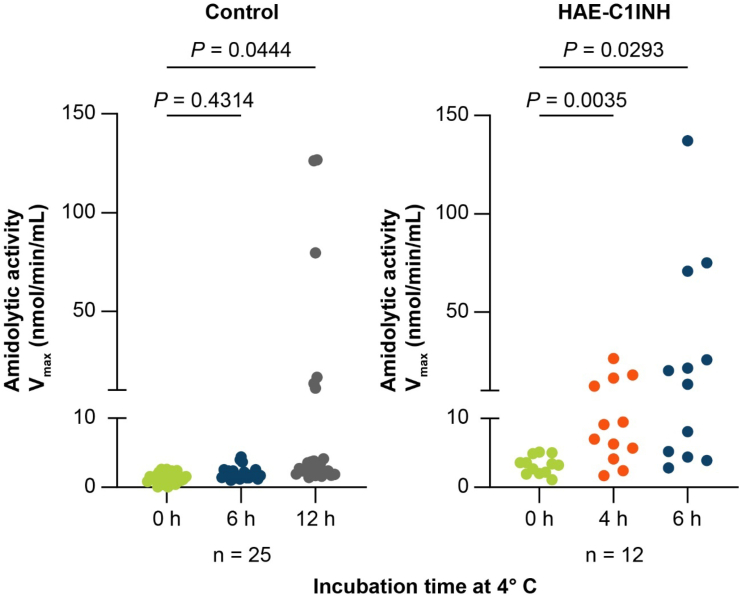
**Effect of cold incubation duration of plasma on total amidolytic activity.** Amidolytic activity (V_max_) was measured at baseline, after 6 h, and 12 h of incubation at 4 °C for samples from volunteers without HAE (controls), and after 4 h and 6 h incubation at 4 °C for samples from patients with HAE-C1INH. HAE-C1INH, hereditary angioedema with decreased levels or activity of C1 inhibitor

Control plasma samples from an initial subset of volunteers without HAE (*n* = 25) were analyzed at baseline and after 6 h and 12 h of cold exposure to determine the optimal time point for subsequent studies. At baseline, total amidolytic activity, i.e., including activity not inhibitable with a PKa inhibitor, in control samples was 1.39 ± 0.15 nmol/min/mL (mean ± SEM). Compared with baseline, no significant increase in total amidolytic activity was observed after 6 h of cold exposure (1.60 ± 0.29 nmol/min/mL, *p* = 0.43), but after 12 h of cold exposure several control samples showed significantly increased total amidolytic activity (16.89 ± 7.29 nmol/min/mL, *p* = 0.044) (Fig. 1).

Plasma samples from a subset of randomly selected patients with HAE-C1INH (*n* = 12) were analyzed at baseline and after 4 h and 6 h of cold exposure. At baseline, total amidolytic activity was 3.23 ± 0.38 nmol/min/mL. Mean total amidolytic activity was significantly increased after 4 h of cold exposure (9.89 ± 2.09 nmol/min/mL, *p* = 0.0035) and further increased after 6 h of cold exposure (32.31 ± 11.92 nmol/min/mL, *p* = 0.029) compared with baseline (Fig. 1).

### Measurement of sPKa activity is achieved by reducing non-specific amidolytic activity

**Fig. 2:**
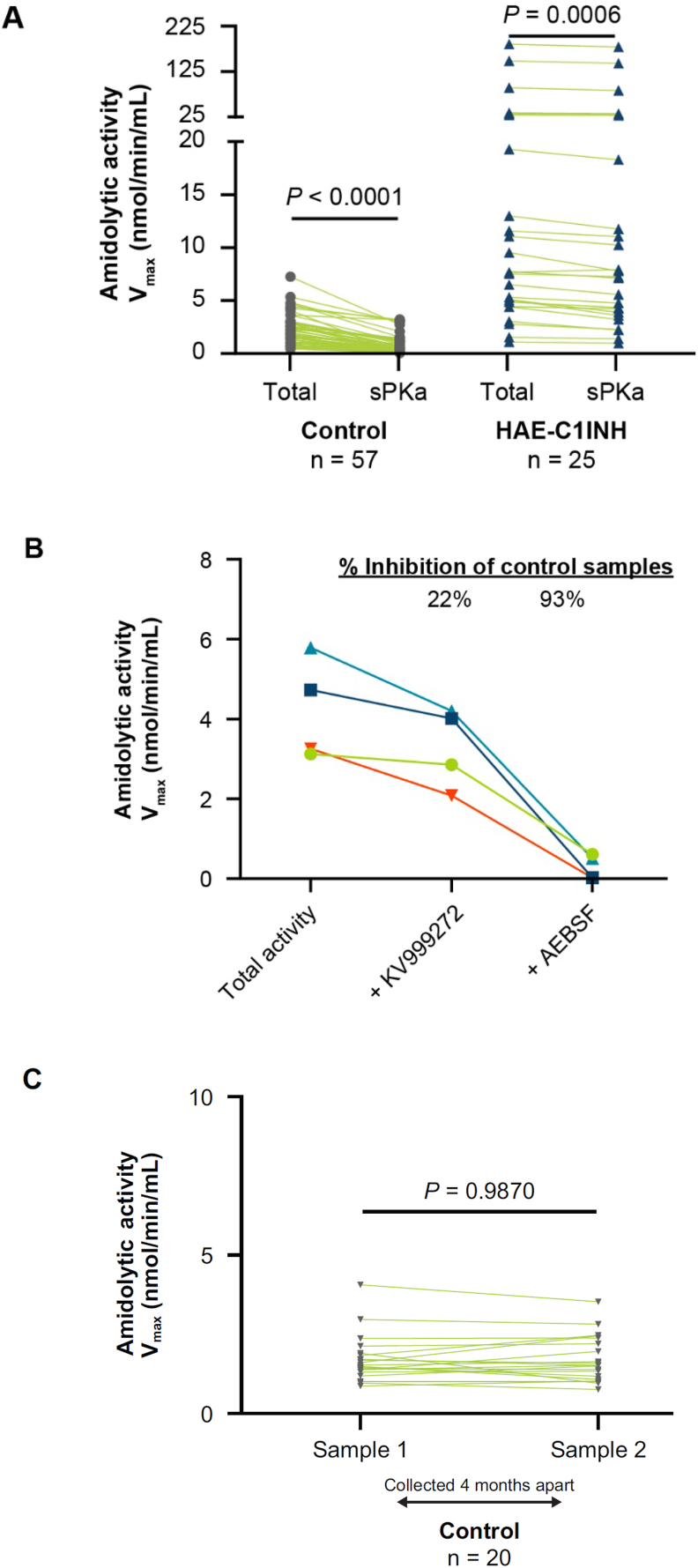
**Characterization of specific PKa activity in plasma.** (A) Comparison of total amidolytic activity and specific plasma kallikrein (sPKa) activity in plasma samples from volunteers without HAE (controls) and patients with HAE-C1INH. (B) Effects of PKa-specific inhibitor KV999272 and broad-spectrum protease inhibitor AEBSF on amidolytic activity in control plasma samples. (C) Analysis of total amidolytic activity in plasma samples collected 4 months apart (Sample 1 and Sample 2) from controls (*n* = 20). AEBSF, 4-(2-aminoethyl) benzenesulfonyl fluoride hydrochloride; HAE-C1INH, hereditary angioedema with decreased levels or activity of C1 inhibitor; PKa, plasma kallikrein; sPKa, specific plasma kallikrein

Total amidolytic activity and sPKa activity were measured after 6 h of cold exposure in 57 control plasma samples from volunteers without HAE and 25 samples with sufficient volume and non-hemolyzed plasma from patients with HAE-C1INH. In control samples, mean ± SEM total amidolytic activity was 2.19 ± 0.19 nmol/min/mL and sPKa activity was 0.88 ± 0.11 nmol/min/mL (*p* < 0.0001). Total amidolytic activity was 25.92 ± 9.24 nmol/min/mL and sPKa activity was 24.53 ± 8.92 nmol/min/mL (*p* = 0.0006) in HAE-C1INH samples (Fig. 2A). This corresponded to a 40% inhibition of total amidolytic activity by the PKa inhibitor KV999272 in control plasma samples and a 95% inhibition in HAE-C1INH samples.

AEBSF was used to test whether the amidolytic activity could be inhibited by a broad-spectrum protease inhibitor in 4 control samples for which <40% of their total amidolytic activity was inhibited by KV999272. These samples were analyzed after 6 h of cold incubation. Results showed that KV999272 inhibited a mean of 22% of the total amidolytic activity in these samples, while AEBSF inhibited a mean of 93% (Fig. 2B).

Variability in amidolytic activity between sample collections was measured using samples from the same volunteers without HAE (*n* = 20) collected at 2 time points 4 months apart (Fig. 2C). No significant difference was observed in the mean amidolytic activity between collection time point 1 (1.72 ± 0.17 nmol/min/mL) and collection time point 2 (1.72 ± 0.16 nmol/min/mL) (*p* = 0.987).

### Cold-induced sPKa activity assay has high assay sensitivity and specificity

**Fig. 3:**
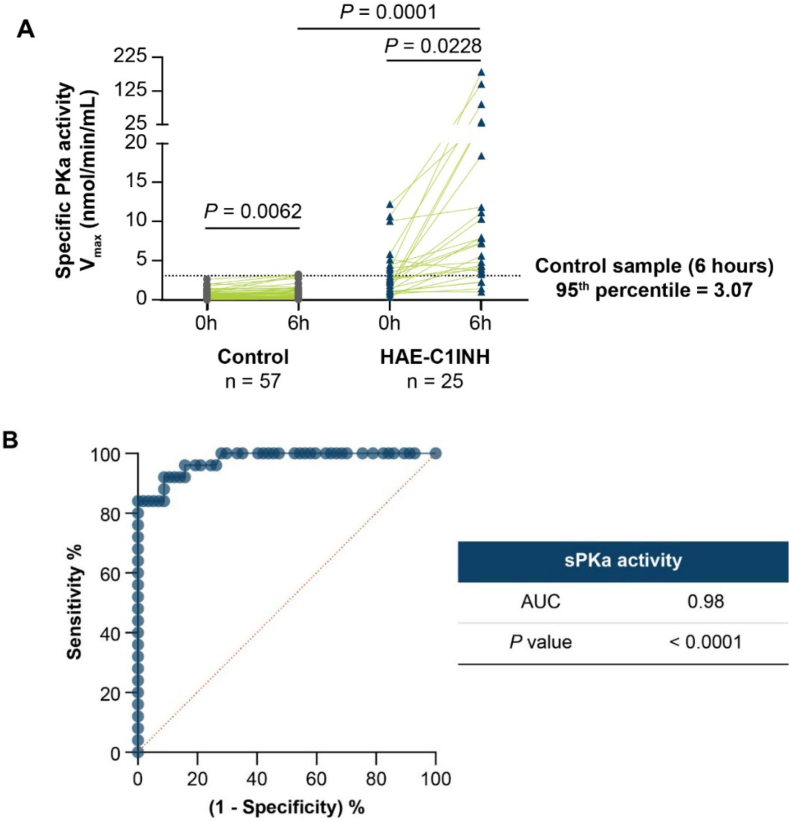
**Cold-induced specific PKa (sPKa) activity assay in volunteers without HAE (controls) and patients with HAE-C1INH.** (A) sPKa activity in plasma samples from controls (*n* = 57) and patients with HAE-C1INH (*n* = 25). sPKa activity was measured at baseline and after 6 h of incubation at 4 °C. A threshold was set at the 95th percentile of sPKa activity in control plasma (3.07 nmol/min/mL) after 6 h of incubation at 4 °C. (B) ROC analysis on the sPKa activity assay, generated using the sPKa activity after 6 h of activation at 4 °C for control and HAE-C1INH samples. AUC, area under the curve; HAE-C1INH, hereditary angioedema with decreased levels or activity of C1 inhibitor; PKa, plasma kallikrein; sPKa, specific plasma kallikrein; ROC, receiver operating characteristic

In control plasma, sPKa activity was 0.69 ± 0.071 nmol/min/mL at baseline and 0.88 ± 0.11 nmol/min/mL after 6 h of cold incubation (*p* = 0.0062). The 95th percentile of cold-induced sPKa activity 6 h in control plasma was 3.07 nmol/min/mL. In plasma from patients with HAE-C1INH, sPKa activity was significantly increased after 6 h of cold incubation. sPKa activity (Fig. 3A) was 3.43 ± 0.64 nmol/min/mL at baseline and 24.53 ± 8.92 nmol/min/mL after cold incubation (*p* = 0.023). After 6 h of cold incubation, sPKa activity in HAE-C1INH plasma was about 28-fold greater than sPKa activity in control plasma (*p* = 0.0001) and sPKa activity in HAE-C1INH plasma samples was above the 95th percentile value for control plasma with assay specificity of 95% and sensitivity of 84%. The AUC of the ROC analysis curve (Fig. 3B) was 0.98 (*p* < 0.0001).

### sPKa activity is elevated in plasma from patients with HAE-nC1INH

**Fig. 4:**
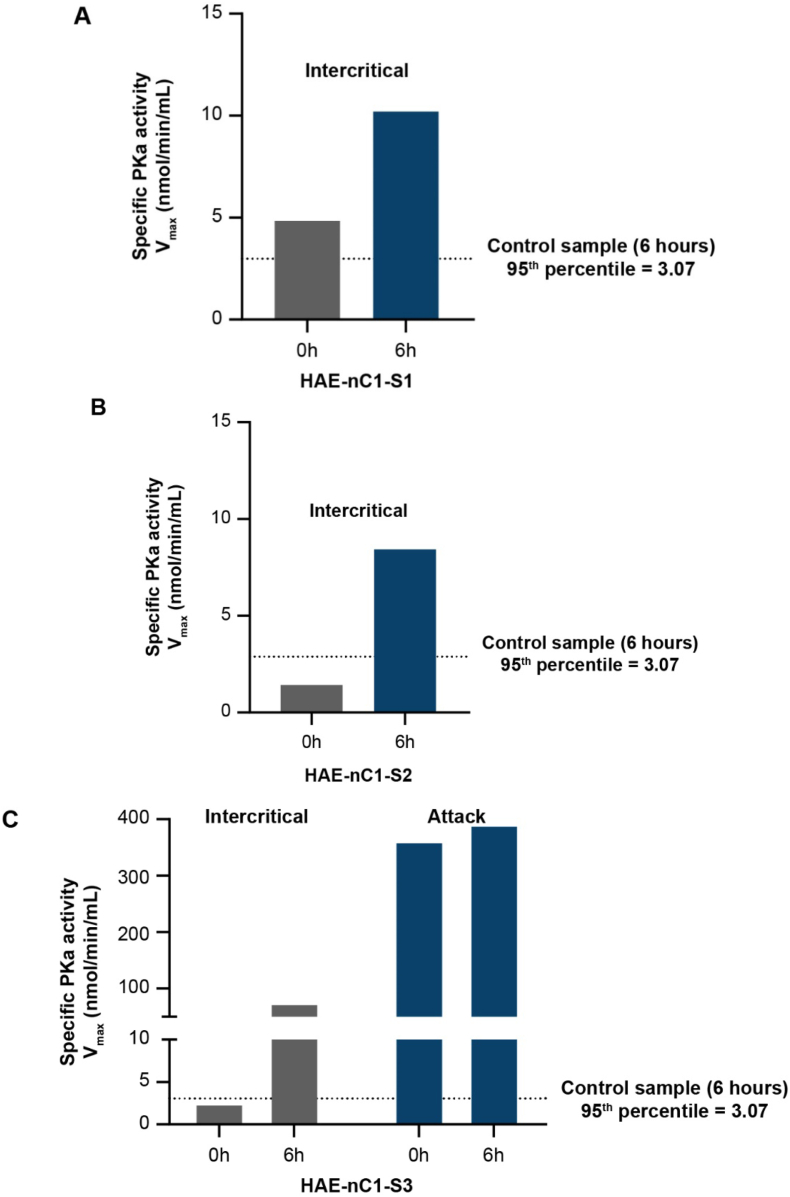
**sPKa activity in plasma samples from individuals diagnosed with HAE-nC1INH.** Samples from HAE-nC1-S1 (A) and HAE-nC1-S2 (B) were collected during the intercritical period. (C) Two samples were collected from HAE-nC1-S3: 1 during the intercritical period, and 1 during an attack. HAE-nC1INH, hereditary angioedema with normal levels and activity of C1 inhibitor; HAE-nC1-S1, patient 1 with HAE-nC1INH; HAE-nC1-S2, patient 2 with HAE-nC1INH; HAE-nC1-S3, patient 3 with HAE-nC1INH; PKa, plasma kallikrein

Plasma samples from 3 individuals with a clinical diagnosis of HAE-nC1INH (HAE-nC1-S1, HAE-nC1-S2, and HAE-nC1-S3) were evaluated in the cold-induced sPKa activity assay. For HAE-nC1-S1, sPKa activity was analyzed using a plasma sample obtained during the intercritical period. sPKa activity in this sample was 4.83 nmol/min/mL at baseline and 10.18 nmol/min/mL after 6 h of cold incubation (Fig. 4A). For HAE-nC1-S2, sPKa activity was also analyzed in a plasma sample collected during the intercritical period. sPKa activity in this sample was 1.42 nmol/min/mL at baseline and 8.43 nmol/min/mL after 6 h of cold incubation (Fig. 4B). For HAE-nC1-S3, 2 plasma samples were collected: 1 during the intercritical period, and 1 during an HAE attack. In the intercritical sample, sPKa activity was 2.48 nmol/min/mL at baseline and 74.33 nmol/min/mL after 6 h of cold incubation. In the sample collected during an HAE attack, sPKa activity was elevated to 361.67 nmol/min/mL at baseline and 390.82 nmol/min/mL after 6 h of cold incubation (Fig. 4C). sPKa activity for all 3 HAE-nC1INH samples after 6 h of cold incubation was greater than the 95th percentile for control samples of 3.07 nmol/min/mL shown in Fig. 3A.

## Discussion

The development of a sensitive and specific assay for measuring PKa activity may enable characterization of the KKS in HAE-C1INH, HAE-nC1INH, and other KKS-mediated diseases as well as predict the response of these diseases to current and future treatment options. In this study, we developed one such assay that detects low levels of PKa activity in plasma samples without the need for highly specialized reagents and instrumentation. This assay was based on the method previously described by Defendi et al.^35^ and was optimized to detect low levels of PKa activity in plasma, including by 1) using a selective PKa inhibitor to enable the measurement of amidolytic activity specifically mediated by PKa, and 2) incubation of plasma at 4^o^ C to induce or amplify PKa activity without the addition of exogenous reagents, such as dextran sulfate, ellagic acid, FXIIa, or silica. Using this sPKa assay, we demonstrated that sPKa activity is elevated during the intercritical period in plasma samples from individuals with HAE-C1INH, with 84% sensitivity and 95% specificity for differentiation from sPKa activity in plasma from volunteers without HAE, when cold incubated for 6 h. In addition, we showed that sPKa activity in plasma from individuals diagnosed with HAE-nC1INH, whether collected during the intercritical period or during an HAE attack, was higher than control samples following cold incubation.

In addition to samples from volunteers without HAE, this study utilized HAE plasma samples collected in the pre-dose period from a phase 2 clinical study of the PKa inhibitor sebetralstat for the on-demand treatment of HAE attacks^41^ and 3 patients with presumed HAE-nC1INH. Because blood sampling and processing can exogenously activate the KKS,^42^ we strictly controlled pre-analytical conditions. Additionally, we excluded samples obtained from females who were receiving oral contraception or hormone replacement therapy since orally-administered estrogen can increase KKS activation.^44^, ^45^, ^46^ The use of plasma from patients with HAE from the phase 2 study enabled the measurement of sPKa activity in a patient cohort that was well defined regarding their baseline attack frequency and prophylactic treatment washout period.^41^

Substrates commonly used in PKa assays, including H-D-Pro-Phe-Arg-*p*NA, can be cleaved by multiple plasma proteases,^47^^,^^48^ which contribute to signal generation that is not mediated by PKa. Therefore, sPKa was quantified by subtraction of amidolytic activity that occurred in the presence of a specific PKa inhibitor from the total amidolytic activity. We showed that only 40% of the amidolytic activity in plasma from control individuals was inhibited by the PKa inhibitor KV999272,^43^ revealing that a substantial fraction of the amidolytic activity in control plasma is due to enzymes other than PKa. In contrast, 95% of the amidolytic activity in HAE-C1INH samples was inhibited by KV999272 and was therefore attributable to PKa activity. Amidolytic activities that were not inhibited by KV999272 were similar in control and HAE plasma (1.3 and 1.4 nmol/min/mL, respectively). The amidolytic activity in control samples that was not inhibited by the PKa inhibitor was inhibited using a broad-spectrum protease inhibitor AEBSF, thereby confirming the contributions of other proteases. Excluding amidolytic activity mediated by enzymes other than PKa improved the sensitivity of the assay to detect small increases in PKa activity in samples from patients with HAE compared with controls.

Since PKa activity in plasma is normally very low, we sought to use a method to activate the KKS without the addition of exogenous reagents, such as dextran sulfate or silica, which are strong activators of the KKS with effects that are difficult to control and can overwhelm endogenous mechanisms of KKS regulation. Previous studies have shown that amidolytic activity, presumed to be PKa, was observed in HAE-C1INH and HAE-nC1INH plasma following incubation at 4^o^ C, whereas little or no activity was detected in plasma from controls without HAE.^39^^,^^45^ In addition, exposure of HAE-nC1INH plasma to cold for 12 h resulted in the cleavage of PK and HK, indicating KKS activation.^45^ These findings suggested that cold exposure provides an opportunity to induce or amplify PKa activity in HAE samples while having minimal effect on control plasma. Using a time course, we show that 6 h of cold exposure was the optimal time point for increasing PKa activity in HAE-C1INH plasma samples while maintaining low PKa activity in control plasma. Extending cold exposure to 12 h increased PKa activity in some control samples without HAE and thereby reduced specificity for differentiating PKa activity in HAE from controls. Since cold exposure of the plasma reduces the control of C1INH on KKS proteases PKa and FXIIa,^49^^,^^50^ the absence of increased amidolytic activity in the control plasma suggests there was insufficient PKa activity to initiate or amplify contact system activation by cold exposure in nearly all control samples.

In this study, we demonstrated that sPKa activity was increased in 3 patients with a presumed diagnosis of HAE-nC1INH. These individuals were diagnosed with HAE-nC1INH by their physicians based on their clinical profile and their response to treatment with HAE-specific medications. Plasma samples were obtained during the intercritical period and in the absence of prophylactic HAE treatments. Plasma from the 3 patients displayed high sPKa activity following 6 h of cold exposure compared with control plasma from volunteers without HAE. A plasma sample obtained from 1 patient with HAE-nC1INH during an attack displayed increased sPKa activity at both 0 and 6 h of cold exposure. Although the measurement of PKa during an attack could be the most informative marker of KKS-mediated HAE, it is often difficult to obtain blood samples during attacks. Since mechanisms that are independent of PKa have been implicated in causing HAE-nC1INH,^51^^,^^52^ specific measurement of PKa may help guide HAE treatment decisions. PKa activity during the intercritical period could provide a candidate biomarker for KKS activation that can aid in the identification of patients who might benefit from medications that inhibit the KKS for the treatment or prevention of HAE attacks. Measurements of sPKa activity in samples from a larger cohort of individuals with HAE-nC1INH are needed to further characterize this heterogeneous population, especially with regard to the impact of HAE genetic mutations on levels of PKa activity.

Additionally, sPKa measurements in samples from individuals with other types of angioedema, such as mast cell-mediated angioedema or acquired angioedema, are needed to further characterize the potential utility of this assay.

The benefits of this sPKa assay are that any inhibitor that specifically inhibits PKa activity in plasma can potentially be used; it does not require specialized antibodies, instrumentation, or complicated blood collection protocols; measurements of amidolytic activity were highly reproducible in control samples from volunteers without HAE collected 4 months apart. This high assay reproducibility suggests that it has utility for the characterization of PKa activity across multiple conditions, which may aid in the identification and characterization of PKa-mediated disorders. Importantly, we show that sPKa activity is increased during the intercritical period in HAE, which may avoid the need to obtain plasma samples during an attack.

*Limitations:* The evaluation of sPKa activity was performed on a small number of individuals with a presumptive diagnosis of HAE-nC1INH, which is a limitation of this report. These individuals were not fully characterized regarding their attack frequency, clinical responses to KKS-directed therapies, and genetics for mutations associated with HAE. While these data suggest that PKa activity is increased in these patients compared with controls without HAE, longitudinal studies on a larger and fully characterized cohort of HAE-C1INH and HAE-nC1INH patients that are adequately powered to detect differences in PKa activity compared with controls are needed to evaluate the potential of this assay as a biomarker and to evaluate intra-individual variability. Evidence for inter-laboratory reproducibility is limited to a small dataset and does not substitute for formal multicenter validation. Both the control and HAE-nC1INH samples were centrifuged at 2000*g* for 10 min at 20 °C, whereas the HAE-C1INH samples, which were used as a positive control, were centrifuged at 1500*g* for 10 min at 4 °C. Although not studied, it is unlikely that the additional 10 min at 4 °C during centrifugation for HAE-C1INH samples significantly affected sPKa activity that was measured post 6 h incubation on ice. In summary, we developed a specific PKa assay that can detect low levels of PKa activity in plasma and can discriminate between HAE-C1INH and control plasma with high sensitivity and specificity. These findings suggest that sPKa activity in plasma during the intercritical period can provide a candidate biomarker for future research characterizing HAE-C1INH and HAE-nC1INH and may also provide a potential biomarker to guide treatment selection in patients.

## Abbreviations

AEBSF, 4-(2-aminoethyl) benzenesulfonyl fluoride hydrochloride; AUC, area under the curve; C1INH, C1 inhibitor; FXIIa, activated factor XII; HAE, hereditary angioedema; HK, high molecular weight kininogen; KKS, kallikrein-kinin system; PKa, plasma kallikrein; ROC, receiver operating characteristic; SERPIN, serine protease inhibitor; sPKa, specific plasma kallikrein.

## Availability of data and materials

Information on materials used and datasets generated during and/or analyzed during the current study are available from the corresponding author.

## Author contributions

DKL, AG, NM, DV, MD, SLH, EPF: conception and design of the study, or acquisition of data, or analysis and interpretation of data.

MDS, PKA, DMC, AA: interpretation of data.

All authors critically reviewed and revised the manuscript for intellectual content. All authors read and approved the final manuscript. All authors agree to be accountable for the accuracy and integrity of the work.

## Ethics approval

Collection of samples was approved by relevant institutional review board or ethics committees, followed good clinical practice guidelines, observed the Declaration of Helsinki, and followed the guidance of the International Conference on Harmonization. All participants provided written informed consent.

## Consent for publication

All the authors reviewed and approved the manuscript, and agreed to submit it to the World Allergy Organization Journal.

## Declaration of Generative AI and AI-assisted technologies in the writing process

No generative AI or AI-assisted technologies were used in writing or preparing this manuscript.

## Funding information

This study was supported by KalVista Pharmaceuticals, Inc.

## Declaration of competing interests

DKL, NM, and SLH are employees and shareholders of KalVista Pharmaceuticals and are named inventors on a patent application for KalVista Pharmaceuticals. PKA and MDS are employees and shareholders of KalVista Pharmaceuticals. AG has received consulting fees from KalVista Pharmaceuticals and is a named inventor on a patent application for KalVista Pharmaceuticals. DV has received honoraria from BioCryst and Takeda and is a shareholder of Takeda. MD has nothing to disclose. DMC has received consulting fees paid to the institution, honoraria paid to the institution, medical writing support, meeting/travel support, research support, and/or served on advisory boards from KalVista Pharmaceuticals, Astria, BioCryst, CSL Behring, Intellia Therapeutics, Ionis Pharmaceuticals, Otsuka, Pharvaris, and Takeda and has had a leadership role in the HAEi Medical Advisory panel for Central Eastern Europe and Benelux. AA has received grants or contracts, consulting fees, honoraria, and/or meeting/travel support from KalVista Pharmaceuticals, Astria, BioCryst, CSL Behring, Ionis, Pharvaris, Octapharma, and Takeda, and is a research committee member of the Canadian Hereditary Angioedema Network on a voluntary basis. EPF is a former employee of KalVista Pharmaceuticals, a shareholder of KalVista Pharmaceuticals, and a named inventor on a patent application for KalVista Pharmaceuticals.

## References

[1] Busse P.J., Christiansen S.C. (2020). Hereditary angioedema. N Engl J Med.

[2] Longhurst H., Cicardi M. (2012). Hereditary angio-oedema. Lancet.

[3] Nussberger J., Cugno M., Amstutz C. (1998). Plasma bradykinin in angio-oedema. Lancet.

[4] Kaplan A.P., Joseph K. (2014). Pathogenic mechanisms of bradykinin mediated diseases: dysregulation of an innate inflammatory pathway. Adv Immunol.

[5] Maurer M., Magerl M., Betschel S. (2022). The international WAO/EAACI guideline for the management of hereditary angioedema-the 2021 revision and update. Allergy.

[6] Björkqvist J., Sala-Cunill A., Renné T. (2013). Hereditary angioedema: a bradykinin-mediated swelling disorder. Thromb Haemost.

[7] Revak S.D., Cochrane C.G. (1976). The relationship of structure and function in human Hageman factor. The association of enzymatic and binding activities with separate regions of the molecule. J Clin Investig.

[8] Cicardi M., Igarashi T., Rosen F.S., Davis A.E. (1987). 3rd. Molecular basis for the deficiency of complement 1 inhibitor in type I hereditary angioneurotic edema. J Clin Investig.

[9] Stoppa-Lyonnet D., Tosa M., Laurent J. (1987). Altered C1 inhibitor genes in type I hereditary angioedema. N Engl J Med.

[10] Drouet C., Lopez-Lera A., Ghannam A. (2022). SERPING1 variants and C1-INH biological function: a close relationship with C1-INH-HAE. Front Allergy.

[11] Binkley K.E., Davis A. (2000). Clinical, biochemical, and genetic characterization of a novel estrogen-dependent inherited form of angioedema. J Allergy Clin Immunol.

[12] Bork K., Barnstedt S.E., Koch P., Traupe H. (2000). Hereditary angioedema with normal C1-inhibitor activity in women. Lancet.

[13] Adatia A., Boursiquot J.N., Goodyear D. (2024). Real-world outcomes of patients with hereditary angioedema with normal C1-inhibitor function and patients with idiopathic angioedema of unknown etiology in Canada. Allergy Asthma Clin Immunol.

[14] Zuraw B.L., Bork K., Bouillet L. (2025). Hereditary angioedema with normal C1 inhibitor: an updated international consensus paper on diagnosis, pathophysiology, and treatment. Clin Rev Allergy Immunol.

[15] Ariano A., D’Apolito M., Bova M. (2020). A myoferlin gain-of-function variant associates with a new type of hereditary angioedema. Allergy.

[16] Bafunno V., Firinu D., D’Apolito M. (2018). Mutation of the angiopoietin-1 gene (ANGPT1) associates with a new type of hereditary angioedema. J Allergy Clin Immunol.

[17] Bork K., Wulff K., Meinke P. (2011). A novel mutation in the coagulation factor 12 gene in subjects with hereditary angioedema and normal C1-inhibitor. Clin Immunol.

[18] Bork K., Wulff K., Möhl B.S. (2021). Novel hereditary angioedema linked with a heparan sulfate 3-O-sulfotransferase 6 gene mutation. J Allergy Clin Immunol.

[19] Bork K., Wulff K., Rossmann H. (2019). Hereditary angioedema cosegregating with a novel kininogen 1 gene mutation changing the N-terminal cleavage site of bradykinin. Allergy.

[20] Dewald G., Bork K. (2006). Missense mutations in the coagulation factor XII (Hageman factor) gene in hereditary angioedema with normal C1 inhibitor. Biochem Biophys Res Commun.

[21] Kiss N., Barabás E., Várnai K. (2013). Novel duplication in the F12 gene in a patient with recurrent angioedema. Clin Immunol.

[22] Bork K., Wulff K., Steinmüller-Magin L. (2018). Hereditary angioedema with a mutation in the plasminogen gene. Allergy.

[23] Bork K., Wulff K., Witzke G., Hardt J. (2015). Hereditary angioedema with normal C1-INH with versus without specific F12 gene mutations. Allergy.

[24] Bork K., Wulff K., Witzke G. (2023). Gene mutations linked to hereditary angioedema in solitary angioedema patients with normal C1 inhibitor. J Allergy Clin Immunol Pract.

[25] Adatia A., Ritchie B. (2023). Successful use of lanadelumab in a patient with hereditary angioedema with normal C1 inhibitor and negative genetic testing. J Allergy Clin Immunol Glob.

[26] Hioki C., Oda Y., Moriwaki S., Fukunaga A. (2024). Effect of lanadelumab on attack frequency and QoL in Japanese patients with hereditary angioedema:report of five cases. J Dermatol.

[27] Jones D.H., Bansal P., Berstein J. (2022). Clinical profile and treatment outcomes in patients with hereditary angioedema with normal C1 esterase inhibitor. World Allergy Organ J.

[28] Kanarek H.J., Mutschelknaus D.A.S. (2024). Clinical experience with Berotralstat in patients with hereditary angioedema with normal C1-esterase inhibitor: a commented case series. J Asthma Allergy.

[29] Lochbaum R., Trainotti S., Hoffmann T.K., Greve J., Hahn J. (2024). A clinical evaluation of patients with known mutations (plasminogen and factor XII) with a focus on prophylactic treatment. J Dermatol Treat.

[30] Dias de Castro E., Pinhal A.L., Bragança M., Parente Freixo J., Martinho A. (2024). Hereditary angioedema with normal C1-inhibitor: clinical and genetic characterization of 15 Portuguese unrelated families. Ann Allergy Asthma Immunol.

[31] Riedl M.A., Danese M., Danese S., Ulloa J., Maetzel A., Audhya P.K. (2023). Hereditary angioedema with normal C1 inhibitor: US survey of prevalence and provider practice patterns. J Allergy Clin Immunol Pract.

[32] Magerl M., Riedl M.A., Arruda L.K (2025). Global frequency, diagnosis, and treatment of hereditary angioedema with normal C1 inhibitor. J Allergy Clin Immunol Glob.

[33] Hofman Z.L.M., de Maat S., Suffritti C. (2017). Cleaved kininogen as a biomarker for bradykinin release in hereditary angioedema. J Allergy Clin Immunol.

[34] Zhang G., Sexton D.J., Faucette R.R., Qiu Y., Wu J. (2017). 2D-LC-MS/MS to measure cleaved high-molecular-weight kininogen in human plasma as a biomarker for C1-INH-HAE. Bioanalysis.

[35] Defendi F., Charignon D., Ghannam A. (2013). Enzymatic assays for the diagnosis of bradykinin-dependent angioedema. PLoS One.

[36] Gangnus T., Burckhardt B.B. (2020). Improving sensitivity for the targeted LC-MS/MS analysis of the peptide bradykinin using a design of experiments approach. Talanta.

[37] Sexton D., Faucett R., Rivera-Hernandez M. (2024). A novel assay of excess plasma kallikrein-kinin system activation in hereditary angioedema. Front Allergy.

[38] Lara-Marquez M.L., Christiansen S.C., Riedl M.A., Herschbach J., Zuraw B.L. (2018). Threshold-stimulated kallikrein activity distinguishes bradykinin- from histamine-mediated angioedema. Clin Exp Allergy.

[39] Larrauri B., Hester C.G., Jiang H. (2020). sgp120 and the contact system in hereditary angioedema: a diagnostic tool in HAE with normal C1 inhibitor. Mol Immunol.

[40] Christiansen S.C., Zuraw B.L. (2024). Contact system activation and bradykinin generation in angioedema: laboratory assessment and biomarker utilization. Immunol Allergy Clin.

[41] Aygören-Pürsün E., Zanichelli A., Cohn D.M. (2023). An investigational oral plasma kallikrein inhibitor for on-demand treatment of hereditary angioedema: a two-part, randomised, double-blind, placebo-controlled, crossover phase 2 trial. Lancet.

[42] Gangnus T., Burckhardt B.B. (2022). Reliable measurement of plasma kinin peptides: importance of preanalytical variables. Res Pract Thromb Haemost.

[43] Clermont A., Murugesan N., Zhou Q. (2016). Plasma kallikrein mediates vascular endothelial growth factor-induced retinal dysfunction and thickening. Investig Ophthalmol Vis Sci.

[44] Birmingham J.M., Wisnivesky J., Busse P.J. (2024). The effect of estrogen-containing birth control pills on the constituents of bradykinin expression in plasma. J Allergy Clin Immunol Glob.

[45] Larrauri B., Hester C.G., Jiang H. (2021). Analysis of cold activation of the contact system in hereditary angioedema with normal C1 inhibitor. Mol Immunol.

[46] Strandberg J., Gade I.L., Palarasah Y. (2023). Combined oral contraceptives may activate the contact system in healthy women. Res Pract Thromb Haemost.

[47] Peng Q., McEuen A.R., Benyon R.C., Walls A.F. (2003). The heterogeneity of mast cell tryptase from human lung and skin. Eur J Biochem.

[48] Takayama T.K., McMullen B.A., Nelson P.S., Matsumura M., Fujikawa K. (2001). Characterization of hK4 (prostase), a prostate-specific serine protease: activation of the precursor of prostate specific antigen (pro-PSA) and single-chain urokinase-type plasminogen activator and degradation of prostatic acid phosphatase. Biochemistry.

[49] Favier B., Bicout D.J., Baroso R., Paclet M.H., Drouet C. (2022). *In vitro* reconstitution of kallikrein-kinin system and progress curve analysis. Biosci Rep.

[50] Patston P.A., Gettins P., Beechem J., Schapira M. (1991). Mechanism of serpin action: evidence that C1 inhibitor functions as a suicide substrate. Biochemistry.

[51] Dickeson S.K., Kumar S., Sun M.F. (2024). A mechanism for hereditary angioedema caused by a methionine-379-to-lysine substitution in kininogens. Blood.

[52] Hintze S., Möhl B.S., Beyerl J. (2022). Mutant plasminogen in hereditary angioedema is bypassing FXII/kallikrein to generate bradykinin. Front Physiol.

